# Environmental Bovine Mastitis Pathogens: Prevalence, Antimicrobial Susceptibility, and Sensitivity to *Thymus vulgaris* L., *Thymus serpyllum* L., and *Origanum vulgare* L. Essential Oils

**DOI:** 10.3390/antibiotics11081077

**Published:** 2022-08-09

**Authors:** Dragana Tomanić, Biljana Božin, Nebojša Kladar, Jovan Stanojević, Ivana Čabarkapa, Nebojša Stilinović, Jelena Apić, Dragana D. Božić, Zorana Kovačević

**Affiliations:** 1 Department of Veterinary Medicine, Faculty of Agriculture, University of Novi Sad, Trg Dositeja Obradovica 8, 21000 Novi Sad, Serbia; 2 Center for Medical and Pharmaceutical Investigations and Quality Control, Department of Pharmacy, Faculty of Medicine, University of Novi Sad, Hajduk Veljkova 3, 21000 Novi Sad, Serbia; 3 Institute of Food Technology, University of Novi Sad, Bulevar Cara Lazara 1, 21000 Novi Sad, Serbia; 4 Department of Pharmacology, Toxicology and Clinical Pharmacology, Faculty of Medicine, University of Novi Sad, Hajduk Veljkova 3, 21000 Novi Sad, Serbia; 5 Scientific Veterinary Institute Novi Sad, Rumenački put 20, 21000 Novi Sad, Serbia; 6 Department of Microbiology and Immunology, Faculty of Pharmacy, University of Belgrade, Vojvode Stepe 450, 11221 Belgrade, Serbia

**Keywords:** antimicrobial resistance, cows, essential oils, mastitis, *Proteus mirabilis*, *Serratia marcescens*

## Abstract

Mastitis is considered to be one of the most important diseases of dairy cows in terms of health, production, and economy. Being the most common cause of antibiotic consumption in dairy cows, treatment of this disease is one of the biggest challenges in the veterinary profession as an increasing number of pathogens develop resistance to antibiotics used in the treatment. Therefore, new alternative approaches for limiting the use of antibiotics in livestock are required. For this reason, our study aimed to investigate prevalence of environmental mastitis associated bacterial strains, as well as the sensitivity of isolated strains to different antibiotics. Additionally, the therapeutic potential of three essential oils (EOs) was tested against bovine *Serratia* spp. and *Proteus* spp. mastitis pathogens, based on their chemical composition, as well as antibacterial potential. The study was carried out on 81 milk samples collected from dairy cows with mastitis. In order to determine prevalence of *S. marcescens* and *P. mirabilis*, microbiological isolation and identification were performed. Antimicrobial susceptibility testing was performed by disk diffusion method and the microdilution method was used to determine the antibacterial activity of selected EOs. In the oregano EO, a total of 23 compounds were detected, with carvacrol as a dominant component (78.94%). A total of 26 components were present in the EO of common thyme, where thymol was the most abundant compound (46.37%). Thymol also dominated (55.11%) the wild thyme EO. All tested EOs displayed antibacterial activity against all strains to different extents, while wild and common thyme EOs were the most effective. It could be concluded that the tested EOs represent promising therapeutic candidates for effective non-antibiotic treatment of mastitis.

## 1. Introduction

Udder in good health condition is an important element in the production of safe-quality milk with proper biological characteristics, since the mammary gland affected by inflammatory processes changes the quality and quantity of milk yield [1]. Mastitis is an inflammatory mammary gland condition of considerable interest due to its high incidence and extensive cost. As the most frequent infection in dairy cows, mastitis can be caused by bacteria, fungi, molds, and algae [2].

Microorganisms that cause mastitis are generally classified as either contagious or environmental based upon their primary reservoir and mode of transmission. The predominant contagious pathogens are *Staphylococcus aureus, Streptococcus agalactiae,* and *Corynebacterium bovis,* while *Escherichia coli, Streptococcus uberis,* and *Streptococcus dysgalactiae* are the predominant environmental pathogens [3,4]. Besides the pathogens mentioned above, *Serratia marcescens* and *Proteus mirabilis* are also grouped as environmental causative agents of mastitis [5,6]. Moreover, in recent times, there has been clear evidence of an increasing incidence of environmental mastitis, while the incidence of contagious mastitis has decreased [7].

Besides causing bovine mastitis, *S. marcescens* is important as a causal agent of reproductive tract infections in cows [8]. Sources of *S. marcescens* infection are diverse due to the prevalence of this pathogen in the environment, but the causative agent can be transmitted from cow to cow, as well as from equipment and accessories for the husband to the cow [8,9]. Furthermore, *S. marcescens* has been reported to cause both clinical and subclinical mastitis outbreaks in dairy cows [10]. According to some data, dairy cows may have subclinical pathogen carriage for several months or even years [10], while infections usually result in changes in the color and consistency of milk [11]. Moreover, some cases of the subclinical form of mastitis can cure spontaneously, but infected cows can also become chronic carriers of *S. marcescens* [10].

Being the most prevalent *Proteus* species, *Proteus mirabilis* is a common opportunistic pathogen causing severe illness in animals [12,13]. As a ubiquitous environmental microorganism widely present in nature [13,14], it can be found in animal breeding facilities and contaminated water, food, vegetables, utensils, equipment, and surgical instruments [15]. Interestingly, *Proteus* spp. commonly contaminate drop hoses used to wash udders before milking [16]. However, *Proteus* infections in domestic animals are often misdiagnosed or considered contaminants rather than a primary agent of disease [15].

Mastitis is by far the most important reason for antibiotic treatment [17]. However, antibiotic resistance in bacteria is an increasing threat, and there is a need for new, alternative therapies, including essential oils (EOs) [18]. Along with other advantages as an alternative to antibiotics, EOs show antibacterial properties and no resistance has been reported after prolonged exposure [19]. There are many in vitro studies for evaluating the antimicrobial efficacy of EOs against common mastitis-associated pathogens [20,21,22,23,24]. The greatest antimicrobial effect is exerted by EOs with thymol and carvacrol as main components [25,26]. To the best of our knowledge, there is only one report about antimicrobial activity of major EOs’ components cinnamaldehyde and carvacrol on *P. mirabilis* as foodborne microorganisms [27]; no one has focused on these two causative agents of mastitis, although they are important in development of this disease. Furthermore, the prevalence and antibiotic susceptibility of *P. mirabilis* and *S. marcescens* have not been studied in Serbia so far.

Therefore, the present study aimed to evaluate the prevalence and antibiotic susceptibility pattern of the mastitis causing *P. mirabilis* and *S. marcescens* in dairy herds. Additionally, the effectiveness of selected EOs (*Origanum vulgare* L., *Thymus serpyllum* L., and *Thymus vulgaris* L.) from the *Lamiaceae* family against isolates of these two bacterial species was evaluated.

## 2. Results

### 2.1. Bacteriological Testing of Milk Samples

**Figure 1:**
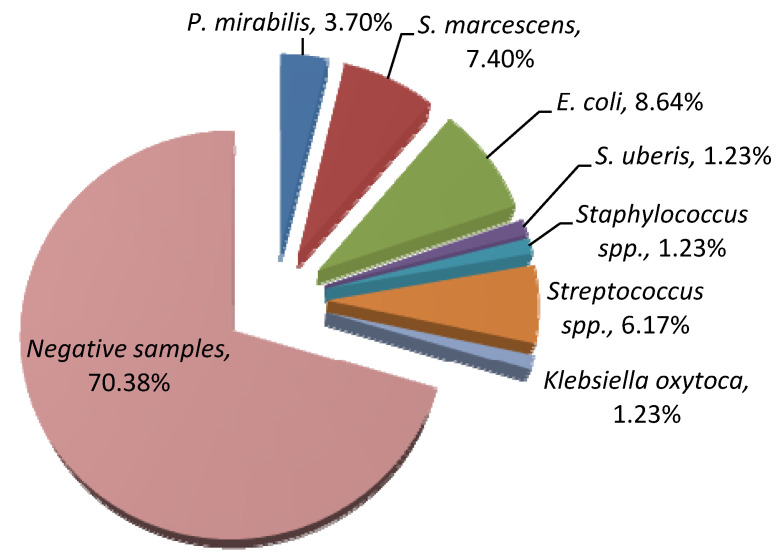
Prevalence of mastitis-causing pathogens in the collected milk samples.

Based on laboratory results, out of a total of 81 milk samples, 24 (29.62%) were positive for mastitis-causing pathogens, while the remaining 57 samples (70.38%) were negative in bacteriological tests. In addition, milk samples were collected from cows with diagnosed subclinical and clinical mastitis, totaling 15 and 9 cases, retrospectively. Of the isolated bacteriological causes of mastitis, *P. mirabilis* was isolated in 3 cases (3.7%), out of which 2 were subclinical and 1 was clinical mastitis. *S. marcescens* was present in 6 samples (7.4%), out of which 4 were subclinical and 2 were clinical mastitis. Figure 1 illustrates the prevalence of mastitis pathogens in tested samples.

### 2.2. Chemical Composition of Selected EOs

Chemical composition of the EOs of common (*Thymus vulgaris* L.) and wild thyme (*Thymus serpyllum* L.) as well as oregano (*Origanum vulgare* L.) are listed in Table 1. A total of 23 compounds were detected in oregano EO, representing 99.13% of mixture. The main class of compounds were aromatic oxygenated monoterpenes (51.70%) with carvacrol as a dominant component (78.94%). Furthermore, a notable content of thymol (4.87%) and *p*-cymene (4.52%) was recorded. A total of 26 components were present in the EO of common thyme, representing 99.27% of identified compounds. As in the EO of oregano, in thyme EO aromatic oxygenated monoterpenes were the main class of compounds (51.7%), followed by aromatic monoterpene hydrocarbons (23.83%) and monoterpene hydrocarbons (11.26%). The most abundant compounds were thymol (46.37%), *p*-cymene (23.83%), *γ*-terpinene (3.46%), and *trans*-β-caryophyllene (3.86%). Similar compounds were identified in the EO of wild thyme, where a total of 28 compounds (99.93%) were identified. The main class of compounds was aromatic oxygenated monoterpenes (55.78%). Furthermore, monoterpene hydrocarbons (25.78%) and aromatic monoterpene hydrocarbons (16.66%) were detected in a notable amount. The dominant compound was thymol (55.11%), followed by *γ*-terpinene (22.31%) and *p*-cymene (16.66%).

### 2.3. Antibiotic Susceptibility Testing of Mastitis-Associated Bacteria

The results of antibiotic susceptibility testing of the bacterial isolates are shown in Table 2. *P. mirabilis* and *S. marcescens* isolates were the most susceptible to gentamicin, enrofloxacin, ceftriaxone, and neomycin (100% each). Overall, 85.75% of the bacterial isolates were susceptible to trimethoprim/sulfamethoxazole, followed by 71.42% susceptibility to streptomycin. On the contrary, full resistance was noted against cloxacillin, novobiocin, penicillin, and ampicillin (100%), followed by lincomycin (85.71%), and tetracycline and erythromycin (71.42%).

**Figure 2:**
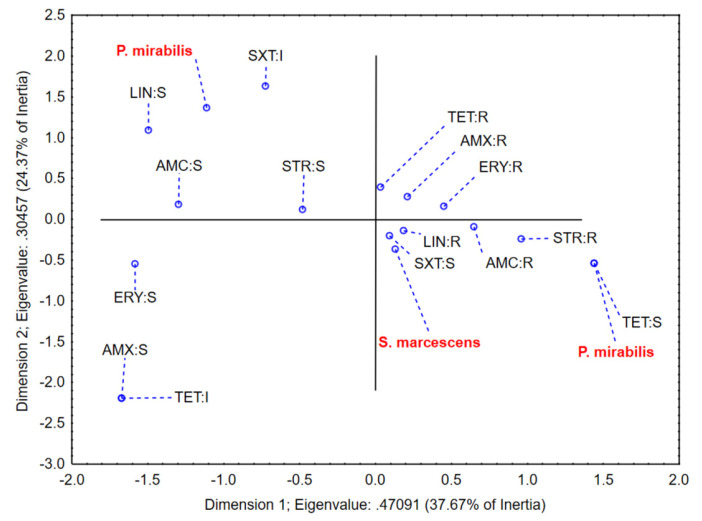
The position of the evaluated variables in the space defined by the first two correspondent axes.

The application of multiple correspondence analysis on a dataset describing susceptibility of *P. mirabilis* and *S. marcescens* to applied antimicrobial agents (except those acting completely effective or ineffective in case of all isolates; thus, showing no data variability) shows that the first two correspondent axes describe more than 60% of samples’ variability. The close grouping of *S. marcescens* isolates in the positive part of the first correspondent axis with resistance to tetracycline, amoxycillin, erythromycin, and lincomycin, as well as sensitivity to the trimethoprim/sulfamethoxazole combination can be noticed (Figure 2). On the other hand, *P. mirabilis* isolates are located in the negative as well as in the positive part of the first correspondent axis, since significant variations regarding susceptibility to lincomycin, amoxicillin/clavulanic acid, trimethoprim/sulfamethoxazole, streptomycin, and tetracycline could be observed, suggesting a potentially emerging pathogen regarding antimicrobial resistance.

### 2.4. EOs Effectiveness against Mastitis-Associated Bacteria

To evaluate the antimicrobial activity of selected EOs against *P. mirabilis* and *S. marcescens* strains, the determinations of minimum inhibitory concentrations (MICs) and minimal bactericidal concentrations (MBCs) were performed. The results presented in Table 3 showed the variable effects of EOs on the tested bacterial strains. MIC values for EOs against *P. mirabilis* strains were 3.125 mg/mL and MBC values ranged between 3.125 and 6.25 mg/mL. On the other hand, EOs of common and wild thyme had low MIC values for *S. marcescens* isolates (MIC = 1.56 mg/mL). Our results demonstrated that oregano EO showed a lower MBC value for *P. mirabilis* compared with *S. marcescens*.

**Figure 3:**
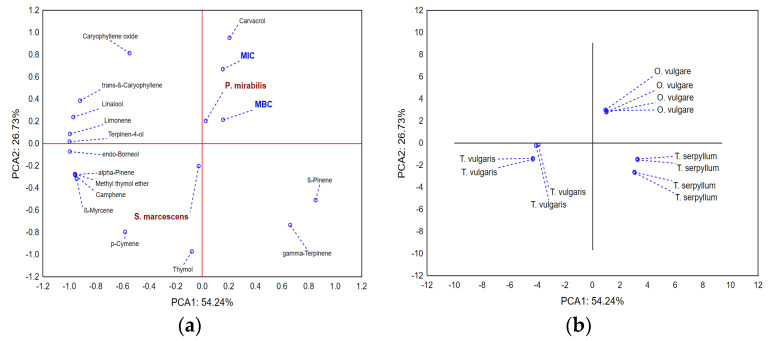
Antimicrobial potential and chemical profiles of the essential oils—compounds. PCA loadings (**a**) and positions of the evaluated samples in the space defined by the first two principal components’ axes (**b**).

The application of Principal components analysis (PCA) on dataset describing minimum inhibitory concentrations (MICs) and minimal bactericidal concentrations (MBCs) of evaluated EOs, as well as the chemical composition of the EOs (compounds abundant more than 1%), shows that the first two principal components describe more than 80% of the samples’ variability (Figure 3a). In terms of the first principal component (PCA1), most of the variability is described by the presence of β-pinene, linalool, limonene, terpinene-4-ol, and endoborneol, while the shape of variability mostly correlates (PCA2) with the amounts of carvacrol, thymol, as well as the recorded MIC values. The positions of the evaluated EOs in the space defined by the first two principal component axes (Figure 3b) show that *O. vulgare* EO is the least effective against both pathogens, while *Thymus* sp. EOs show similar activity. Furthermore, *Thymus* sp. EOs are characterized by higher abundance of thymol, while carvacrol is more abundant in *O. vulgare* EO. It seems that differences in quantities of β-pinene, linalool, limonene, terpinene-4-ol, and endoborneol do not affect the antimicrobial potential of evaluated *T. vulgaris* and *T. serpyllum* EOs.

**Figure 4:**
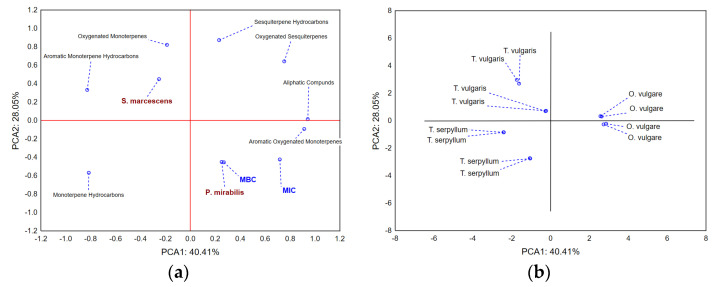
Antimicrobial potential and chemical profiles of the essential oils—classes of compounds. PCA loadings (**a**) and positions of the evaluated samples in the space defined by the first two principal components’ axes (**b**).

Furthermore, the application of PCA on the dataset describing MICs and MBCs of evaluated EOs, as well as the chemical composition of the EOs (classes of compounds abundance), shows that the first two principal components describe around 70% of samples’ variability (Figure 4a). The size of the recorded samples’ variability mostly correlates with the quantified amounts of aliphatic compounds, aromatic oxygenated monoterpenes, aromatic monoterpene hydrocarbons, as well as monoterpene hydrocarbons. The shape of the variability is in terms of the second principal component defined by the presence of oxygenated monoterpenes and sesquiterpene hydrocarbons. The positions of the evaluated EOs in the space defined by the first two principal components’ axes (Figure 4b) show weaker antimicrobial potential of *O. vulgare* EO, which is characterized by the higher presence of aliphatic compounds and aromatic oxygenated monoterpenes. On the other hand, the positions of *Thymus* sp. EOs in the negative part of PCA1 indicate stronger antimicrobial potential and presence of higher amounts of monoterpene hydrocarbons and aromatic monoterpene hydrocarbons, while the two evaluated EOs differ in the amount of oxygenated monoterpenes and sesquiterpene hydrocarbons, which are more abundant in *T. vulgaris* EO.

## 3. Discussion

As mentioned above, mastitis is one of the most important infectious diseases of the dairy industry and is considered to be a great challenge worldwide. Among the predominant environmental pathogens in this disease, the most frequent and major pathogen is *E. coli* [28,29,30]. Our study also revealed that *E. coli* (8.64%) was the major pathogen causing mastitis, followed by *S. marcescens* (7.40%), *Streptococcus* spp. (6.17%), and *P. mirabilis* (3.70%). Furthermore, our research results are similar with other studies since it has been estimated that mastitis-associated *Serratia* spp. account for approximately 9–12% of all naturally acquired Gram-negative bacterial infections. Moreover, *S. marcescens* is one of the most prevalent *Serratia* species [9]. Contrary to our findings, the prevalence of this mastitis-associated pathogen may be even higher than 20% [8,31]. In addition, the prevalence of *S. marcescens* was higher than those reported in China [29], Iraq [32], Brazil [33], and Japan [34]. Interestingly, according to Bannerman et al. [9], the mild clinical symptoms displayed during mastitis—as well as the finding that this pathogen is shed in low numbers—complicates the ability to identify *Serratia* spp. as a causative agent of mastitis during outbreaks.

*P. mirabilis* have been described as the causal agent of bovine mastitis [7,35,36,37]. Our results are similar to the study conducted by Parmar et al. [35], who isolated two *Proteus* spp. samples out of 29 milk samples. These results are close to those reported by other authors [36,37] who reported that the mastitis rate was 2.66% and 2.4%, retrospectively. On the other hand, Verma et al. [38] reported higher prevalence of *P. mirabilis* isolated in cows with mastitis (8.51%). According to Kasa et al. [36] the prevalence of *Proteus* spp. might be due to the residing of this agent in the cow’s environment bedding, feed, and water due to poor environmental sanitation and milking practice.

Antimicrobial treatment of mastitis is anticipated to become problematic in the near future due to the rapid increase in antibiotic-resistant pathogens [39,40]. For this reason, there is a growing need to identify and use alternatives to antibiotics such as natural formulations. Development of such strategies requires detailed evaluation of the chemical composition and antimicrobial potential of EOs.

Although the evaluated EOs of the same manufacturer were used in previous studies [21,22], their chemical composition was evaluated once more, since there could be differences recorded among different series of production. The eventually presented differences could be caused by the origin of raw plant material, place of cultivation or collection (if the plant is collected from nature), as well as a range of ecological factors influencing the content of the main compounds [41,42].

The results obtained in this study regarding oregano EOs’ chemical composition point out carvacrol as a dominant compound, which is in agreement with the previously published data [22,43,44]. Analysis of common and wild thyme EOs’ chemical composition in this study revealed that the content of thymol in the wild thyme EO is higher (55.11%) than in the common thyme EO (46.37%). Unlike thymol, the content of carvacrol is reversed (3.25% in common thyme and 0.67% in wild thyme). These results of EOs’ chemical composition revealed that both tested EOs are in accordance with the requirements prescribed by Ph. Eur. 10 (2020) [45]. The content of total aromatic oxygenated monoterpenes in the EOs of *T. vulgaris* and *T. serpyllum* evaluated in this research is similar to those used in previous research [21] and meets the pharmacopoeia requirements for this class of compounds.

Antimicrobials are an important part of mastitis therapy [40,46,47]. Determination of the antimicrobial susceptibility pattern is necessary to choose the appropriate antimicrobial treatment for cows [48]. *S. marcescens* mastitis is challenging to treat since the isolates from cases of mastitis are reported to easily acquire resistance to various approved antibiotics due to multidrug efflux pump [9,10,34]. In addition, Milanov et al. [8] reported that *S. marcescens* isolated in bovine mastitis showed resistance to penicillin, ampicillin, amoxicillin-clavulanic acid, and tetracycline, which is in agreement with our results. High level of resistance to penicillin, ampicillin, and tetracycline can be associated with the fact that those antibiotics are among the most commonly prescribed in the treatment of mastitis in Serbia [49]. *S. marcescens* isolates exhibited high susceptibility to sulfamethoxazole-trimethoprim in our study, which was also confirmed by Ohnishi et al. [34].

Despite the fact that *S. marcescens* isolates from cow’s milk are usually sensitive in vitro to antibiotics, their use in the treatment of mastitis does not produce satisfactory results and is not recommended. Even in the cases of using the correct treatment, the bacteriological elimination rate is low, with poor responses to antimicrobial therapy [33]. Signs of improvement can be noticed but this condition is mostly temporary, and the cure rate is less than 14% [8,50].

Gentamicin, amoxicillin/clavulanic acid, ceftriaxone, enrofloxacin, and sulfonamides/trimethoprim are considered first-line antimicrobials for treating *Proteus* spp. infections in domestic animals [15]. In the current study, *Proteus* strains showed the highest in vitro sensitivity to the abovementioned antibiotics. Furthermore, *Proteus* spp. resistance to novobiocin, penicillin, amoxycillin, ampicillin, and cloxacillin was observed in this study. The resistance of isolates to novobiocin may be attributed to the prolonged use of these drugs in veterinary medicine since 1950 [15]. In addition, resistance of *Proteus* spp. isolates to cephalosporins and penicillins can be associated with the production of plasmid-mediated beta-lactamase enzymes that promote resistance to some beta-lactams [14]. Interestingly, a high resistance rate for tetracycline was obtained for both strains. Djebala et al. [14] reported that tetracyclines can possess natural antimicrobial resistance. Moreover, gentamicin and enrofloxacin showed great antimicrobial activity against all isolated strains. These antibiotics are newer antimicrobial agents and are less commonly used for treatment of mastitis, resulting in higher efficacy of these drugs [37]. In addition, *Serratia* spp. [10] and *P. mirabilis* [51] can form biofilms, which can also decrease response to the antibiotic treatment.

As presented above, the use of antibiotics is not entirely satisfactory as it can lead to development of resistant strains of bacteria that can impact the therapy outcome. Additionally, the antibiotics may require long-term treatment, which leads to significant economic impacts due to losses in milk production and costs of the antibiotic [52].

To address the concern related to bacterial resistance due to the uncontrolled use of antibiotics in dairy herds, research on alternative treatments is on the rise [53]. Alternative methods, such as EOs, have recently been gaining more attention, and are considered to be a safe, effective, and inexpensive option for the treatment of this disease [54,55].

In the present study, in vitro antibacterial activity of EOs derived from common and wild thyme, and oregano were investigated against isolated mastitis pathogens. To the best of our knowledge, this is the first report regarding the antibacterial activity of EOs against *P. mirabilis* and *S. marcescens* mastitis-associated pathogens. The results of MIC and MBC values showed that selected EOs have antibacterial activity against bovine mastitis isolates. The results obtained in this study indicate that the common and wild thyme EO showed stronger antibacterial in comparison with oregano EO (*p* ≤ 0.05). High antibacterial activity can be related to the presence of higher amounts of compounds, such as carvacrol and thymol, with proven antimicrobial potential [26,56,57]. Kovacevic et al. [21] and Kovacevic et al. [22] observed, in an in vitro study, a strong antibacterial activity of wild thyme and oregano EOs against mastitis-associated pathogens. Velebit et al. [27] presented EO components, carvacrol and cinnamaldehyde, as effective natural antimicrobial agents against foodborne *P. mirabilis.*

However, in vitro activity does not ensure in vivo efficacy because the bovine mammary gland is a difficult target and the penetration of substances when administered parenterally or intramammary depends on the pharmacokinetic characteristics of the drug [46]. So far, our research is the first to combine *Serratia* and *Proteus* bovine mastitis with the antimicrobial potential of EOs, opening new possibilities for their utilization in the treatment and control of bovine mastitis.

## 4. Materials and Methods

### 4.1. Sampling Procedure

The experimental protocol was approved by the Animal Ethics Committee of the Ministry of Agriculture, Forestry and Water Management-Veterinary Directorate (9000-689/2, 06 July 2020). The study was conducted in December 2021 in dairy herd on a farm located in the Vojvodina district, Republic of Serbia. The herd size of the selected farm was 550 cows. A total of 81 Holstein Friesian lactating cows were selected for taking milk samples. The criteria for sampling was the presence of clinical or subclinical form of mastitis, without other health problems. Evaluation of clinical mastitis was performed by veterinarians on the farm, on the basis of clinical udder and milk examination. Clinical mastitis was assessed by clinical examination, while subclinical mastitis was confirmed by the California Mastitis Test using somatic cell count in the milk samples. Clinical symptoms of udder inflammation were swelling, pain, and redness, while changes in the first jets of milk were clots, color change, and density. All cows were hand-milked twice daily, in the morning and evening. Samples for bacteriological testing were taken during morning milking. The udder and teats were washed with water and the teats were disinfected with disinfectant Oxy-Foam^®^ D (Ecolab Hygiene d.o.o., Serbia) and dried with a tissue paper; then, the tip of each teat was disinfected with 70% ethanol, after which milk samples were taken. Milk samples were taken with sterile gloves into sterile bottles. The first few streams of milk were discarded and approximately 10 mL of milk was collected into marked sterile tubes and stored in an ice container at 4 °C during transport to the Laboratory for Milk Hygiene at the Department of Veterinary Medicine, Faculty of Agriculture, University of Novi Sad. For bacteriological isolation, determination, and identification of mastitis pathogens, standard bacteriological diagnostic methods were used [21,22].

### 4.2. Essential Oils

EOs of common (*Thymus vulgaris* L.) and wild thyme (*Thymus serpyllum* L.), as well as oregano (*Origanum vulgare* L.), all belonging to the Lamiaceae family, were evaluated in present study. These EOs are commercially available on the Serbian market and produced in January 2022 by a certified manufacturer (Pharmanais d.o.o., Serbia). All plant raw materials were identified and voucher specimens (F12/2022, F13/2022, F14/2022) were kept at the Herbarium of drugs of the Pharmacognosy and phytotherapy laboratory, Department of Pharmacy, Faculty of Medicine, University of Novi Sad. EOs were obtained by manufacturer using the internal steam distillation technique (Cellkraft AB, Sweden) according to the certificate obtained from the manufacturer.

### 4.3. Essential Oils Chemical Analysis

Chemical composition of investigated EOs was carried out on HP-5MS capillary column (30 m × 0.25 mm; film thickness, 0.25 μm) on Agilent 6890B GC-FID instrument coupled to Agilent 5977 MSD. All samples were injected in split mode (50:1) at an inlet temperature of 220 °C. The starting temperature of the oven was set at 60 °C and increased at a rate of 3 °C/min up to 246 °C. Helium was the carrier gas (1 mL/min) and the temperature of the MSD transfer line was set to 250 °C. Mass spectral data were collected in scan mode (m/z = 50–550). The identification of compounds present in the investigated EOs was performed using the NIST (National Institute of Standards and Technology, Gaithersburg, MD, USA) (v14) mass spectral database and comparison of relative retention indices (RT), as well as literature data [58].

### 4.4. Antibiotic Susceptibility Testing of Mastitis-Associated Bacteria

Antibiotic susceptibility profile of selected bacterial isolates was assessed by in vitro disk diffusion (Kirby–Bauer) method on Mueller–Hinton agar (Oxoid) [59] using six commercially available antibiotic disks (Bioanalyse^®^-Ankara, Turkey) with the following disc potency: ampicillin (10 μg), streptomycin (10 μg), gentamicin (10 μg), trimethoprim/sulfamethoxazole (1.25/23.75 μg), enrofloxacin (5 μg), and ceftriaxone (30 μg). Each isolate was inoculated on nutrient broth and incubated aerobically overnight at 35 ± 1 °C. Bacterial suspension was vortexed and further diluted to the optical density of 0.5 McFarland standard (approximately 1.5 × 10^8^ CFU/mL). The inoculum was spread on the surface of Mueller–Hinton agar with a 10 μL calibrated microbiological loop in three directions in order to achieve confluent bacterial growth. Antibiotic discs were immediately placed on the surface of the agar plate with sterile forceps, and plates were incubated aerobically at 35 ± 1 °C for 18 ± 2 h. The diameters of the zones of inhibition were read from the back of the plate against a dark background illuminated with reflected light. Based on the susceptibility to antimicrobials, the bacteria were categorized into three groups: sensitive (S), intermediate (I), and resistant (R). The interpretation on susceptibility was conducted in accordance with the guidelines of Clinical and Laboratory Standard Institute (CLSI) [60,61].

### 4.5. The Determination of EOs’ Effectiveness against Mastitis-Associated Bacteria

To determine minimal inhibitory concentration (MIC) and minimal bactericidal concentration (MBC) of EOs, broth microdilution assay was performed according to CLSI [62]. EOs were diluted in Muller–Hinton broth (Lab M, International Diagnostics Group Plc, Bury, Lancashire, UK) containing 0.5% Tween 80 (Polyoxyethylene sorbitan monooleate, HiMedia Laboratories Pvt. Ltd., Mumbai, India) for dissolving the EOs, as well as for their dilution to a concentration ranging from 1000 to 0.9 mg/mL. Twenty-microliter aliquots of each tested EO were placed into 96-well plates and aliquots of 160 μL of MHB were added to each well. Afterwards, 20 μL of standardized bacterial suspension was added into each well. The test was performed in a total volume of 200 μL with final EOs’ concentrations ranging from 100 to 0.09 mg/mL, while the final microbial concentration was 5 × 10^5^ CFU/mL. The same tests were performed simultaneously for growth control (MHB + test organism), negative control (MHB + solvent + test organism), and sterility control (MHB + test oil). The plates were incubated at 35 ± 1 °C for 18 ± 2 h. Resazurin solution (0.01%) (Sigma-Aldrich, St Louis, MO, USA) at a volume of 10 μL was added to each well after incubation. Wells without the color change (blue color of resazurin remained unchanged) after the incubation period were scored as above the minimum inhibitory concentration (MIC) value. MIC was defined as the lowest concentration of antimicrobial agent that completely inhibits growth of the organism in the microdilution wells. To determine MBC, referring to the results of the MIC assay, the wells showing complete absence of growth were subcultured. Wells without bacterial growth were identified and 100 μL of the solutions from each well were inoculated in plate count agar plates (Lab M, International Diagnostics Group Plc, Bury, Lancashire, UK) and incubated at 35 ± 1 °C for 18 ± 2 h. MBC was defined as the lowest concentration of the EOs that did not show bacterial growth.

### 4.6. Data Analysis

The obtained data were processed by Microsoft Excel v2019 (Microsoft, Redmond, WA, USA) and Tibco Statistica v13.5 (Tibco, Palo Alto, CA, USA) software packages. The results were analyzed by univariate statistics, as well as by multivariate statistical methods (multiple correspondence analysis and principal components analysis) in order to determine the obtained dataset patterns of variability. Principal component analysis is a dimension reduction statistical technique that enables us to describe large (multidimensional) datasets with a lower number of newly formed summary indices called principal components. The obtained simplified model comes with reduced variability of the initial sample but enables better understanding of the samples’ variability structure. Multiple correspondence analysis can be described as a generalization of principle components analysis, in which the analyzed variables are categorical.

## 5. Conclusions

Insight into the prevalence and antimicrobial susceptibility of rarely explored environmental mastitis-associated pathogens *P. mirabilis* and *S. marcescens* in the specified geographical region could be important for future mastitis control program development. Furthermore, antimicrobial susceptibility of isolated pathogens highlights the need for the development of new therapeutic approaches in bovine mastitis treatment. Moreover, sensitivity of EOs from oregano, and common and wild thyme from *Lamiacae* family to these pathogens imply that proposed EOs may be considered promising natural compounds that could be used to develop a safer and more effective formulation for the mitigation of mastitis. Hence, clinical research of EO-based pharmaceutical formulation in the control of clinical and subclinical forms of mastitis has to be the subject of our future research.

## Figures and Tables

**Table 1:** Chemical composition of evaluated EOs.

Peack No.	Compound	RI *	*O. vulgare*	*T. serpyllum*	*T. vulgaris*
**Monoterpene Hydrocarbons**			**4.04**	**25.78**	**11.26**
1	α-Pinene	937	0.18	0.19	1.47
2	Camphene	952	0.14	0.16	1.83
3	β-Pinene	978	0.67	2.37	0.17
4	β-Myrcene	991	0.24	0.32	1.73
6	α-Phellandrene	1005	0.08	0.11	0.15
7	α-Terpinene	1017	0.45	0.15	0.63
9	Limonene	1030	0.79	0.17	1.82
11	*γ*-Terpinene	1060	1.49	22.31	3.46
**Aromatic Monoterpene Hydrocarbons**			**4.52**	**16.66**	**23.83**
8	*p*-Cymene	1025	4.52	16.66	23.83
**Oxygenated Monoterpenes**			**2.49**	**1.57**	**6.49**
10	1,8-Cineole	1032	0.37	0.17	0.84
12	Linalool	1099	1.08	-	2.14
13	Camphor	1145	0.07	0.57	0.27
14	endo-Borneol	1167	0.39	-	1.73
15	Terpinen-4-ol	1177	0.47	0.11	1.28
16	Isomenthol	1183	-	0.63	-
14	α-Terpineol	1189	0.11	0.01	0.19
20	Carvone	1242	-	-	-
23	Geranyl acetate	1382	-	-	-
24	Bornyl acetate	1285	-	0.08	0.04
**Aromatic Oxygenated Monoterpenes**			**83.81**	**55.78**	**51.7**
18	Isothymol methyl ether	1230	-	-	0.83
19	Methyl thymol ether	1235	-	-	1.25
21	Thymol	1291	4.87	55.11	46.37
22	Carvacrol	1299	78.94	0.67	3.25
**Sesquiterpene Hydrocarbons**			**2.88**	**0.14**	**4.94**
25	α-Cubebene	1351	0.01	-	0.09
26	β-Cubenene	1388	-	-	0.01
27	*trans*-β-Caryophyllene	1419	2.49	0.09	3.86
28	Aromandendrene	1440	-	-	-
29	cis-β-Famesene	1443	-	-	-
30	Humulene	1454	0.11	0.05	0.57
31	allo-Aromandendrene	1461	-	-	-
32	*γ*-Muurolene	1477	-	-	-
33	β-Selinene	1486	-	-	-
34	β-Bisabolene	1509	-	-	-
35	*γ*-Cadinene	1513	-	-	-
36	δ-Cadinene	1524	0.27	-	0.41
**Oxygenated Sesquiterpenes**			**1.37**	**0**	**1.05**
37	Caryophyllene oxide	1581	1.37	-	1.05
**Aliphatic Compunds**			**0.02**	**0**	**0**
5	3-Octanol	994	0.02	-	-
**Total of Identified Compounds**			**99.13**	**99.93**	**99.27**

* Retention indices relative to C9-C24 n-alkanes on the HP 5MS column.

**Table 2:** Antimicrobial sensitivity pattern of *S. marcescens* and *P. mirabilis* strains isolated from cows with mastitis (S—sensitive, I—intermediate, R—resistant). AMX, amoxycillin; AMP, ampicillin; CRO, ceftriaxone; ENR, enrofloxacin; ERY, erythromycin; GEN, gentamicin; LIN, lincomycin; NEO, neomycin; PEN, penicillin; STR, streptomycin; TET, tetracycline; AMC, amoxicillin/clavulanic acid; NB, novobiocin; SXT, trimethoprim/sulfamethoxazole; CLO, cloxacillin.

Bacterial Strain	AMX	AMP	CRO	ENR	ERY	GEN	LIN	NEO	PEN	STR	TET	AMC	NB	SXT	CLO
*P. mirabilis*_1	R	R	S	S	S	S	S	S	R	S	R	S	R	S	R
*P. mirabilis*_2	R	R	S	S	R	S	R	S	R	S	R	S	R	I	R
*P. mirabilis*_3	R	R	S	S	R	S	R	S	R	R	S	R	R	S	R
*S. marcescens*_1	S	R	S	S	S	S	R	S	R	S	I	S	R	S	R
*S. marcescens*_2	R	R	S	S	R	S	R	S	R	R	R	R	R	S	R
*S. marcescens*_3	R	R	S	S	R	S	R	S	R	S	R	R	R	S	R
*S. marcescens*_4	R	R	S	S	R	S	R	S	R	S	R	R	R	S	R
*S. marcescens*_5	R	R	S	S	R	S	R	S	R	R	R	R	R	S	R
*S. marcescens*_6	R	R	S	S	R	S	R	S	R	S	R	R	R	S	R

**Table 3:** Minimum inhibitory concentrations (MICs) and minimal bactericidal concentrations (MBCs) of selected EOs against *P. mirabilis* and *S. marcescens.*

Sample(mg/mL)	TS **Average ± SD		TV ***Average ± SD		OV ****Average ± SD	
	MIC	MBC	MIC	MBC	MIC	MBC
*P. mirabilis*	3.125 ± 1.35	6.25 ± 2.7	3.125 ± 0.00	6.25 ± 2.7	3.125 ± 1.35	3.125 ± 1.35
*S. marcescens*	1.56 ± 0.96 *	3.125 ± 1.91 *	1.56 ± 0.96 *	3.125 ± 1.91 *	3.125 ± 1.91	6.25 ± 3.83

* Statistical significance of EOs’ antimicrobial effect between *P. mirabilis* and *S. marcescens* (*p* ≤ 0.05). ** TS—*T. serpyllum* EO; *** TV—*T. vulgaris* EO; **** OV—*O. vulgare* EO.

## Data Availability

The data used to support the findings of this study are available in the present manuscript.

## References

[1] Rogožarski D., Dimitrijević G., Dobrosavljević I. (2012). Participation of Diagnosed Mastitits in Cows in Milk Hygiene of Branicevo District in 2002. Arch. Vet.-Med..

[2] Benić M., Maćešić N., Cvetnić L., Habrun B., Cvetnić Ž., Turk R., Đuričić D., Lojkić M., Dobranić V., Valpotić H. (2018). Bovine mastitis: A persistent and evolving problem requiring novel approaches for its control—A review. Vet. Arh..

[3] Dufour S., Labrie J., Jacques M. (2019). The Mastitis Pathogens Culture Collection. Microbiol. Resour. Announc..

[4] Gomes F., Saavedra M.J., Henriques M. (2016). Bovine mastitis disease/pathogenicity: Evidence of the potential role of microbial biofilms. Pathog. Dis..

[5] Bogni C., Odierno L., Raspanti C., Giraudo J., Larriestra A., Reinoso E., Lasagno M., Ferrari M., Ducrós E., Frigerio C., Mendez-Vilas A. (2011). War against mastitis: Current concepts on controlling bovine mastitis pathogens. Science against Microbial Pathogens: Communicafing Current Research and Technological Advances.

[6] Manrique L.E.T., Villate-Hernández J.R., Andrade-Becerra R.J. (2019). Bacterial and fungal infectious etiology causing mastitis in dairy cows in the highlands of Boyacá (Colombia). Rev. Fac. Med. Vet.-Zootec..

[7] Cervinkova D., Vlkova H., Borodacova I., Makovcova J., Babak V., Lorencova A., Vrtkova I., Marosevic D., Jaglic Z. (2013). Prevalence of mastitis pathogens in milk from clinically healthy cows. Vet. Med..

[8] Milanov D., Prunić B., Košarčić S., Potkonjak A. (2012). Less Common Aetiological Agent of Bovine Mastitis: -*Serratia marcescens*-. Arch. Vet. Med..

[9] Bannerman D.D., Paape M.J., Goff J.P., Kimura K., Lippolis J.D., Hope J.C. (2004). Innate immune response to intramammary infection with *Serratia marcescens* and *Streptococcus uberis*. Vet.-Res..

[10] Friman M.J., Eklund M.H., Pitkälä A.H., Rajala-Schultz P.J., Rantala M.H.J. (2019). Description of two *Serratia marcescens* associated mastitis outbreaks in Finnish dairy farms and a review of literature. Acta Vet. Scand..

[11] Pinzón-Sánchez C., Ruegg P. (2011). Risk factors associated with short-term post-treatment outcomes of clinical mastitis. J. Dairy Sci..

[12] Drzewiecka D. (2016). Significance and Roles of *Proteus* spp. Bacteria in Natural Environments. Microb. Ecol..

[13] Algammal A.M., Hashem H.R., Alfifi K.J., Hetta H.F., Sheraba N.S., Ramadan H., El-Tarabili R.M. (2021). atpD gene sequencing, multidrug resistance traits, virulence-determinants, and antimicrobial resistance genes of emerging XDR and MDR-Proteus mirabilis. Sci. Rep..

[14] Djebala S., Evrard J., Gregoire F., Bayrou C., Gille L., Eppe J., Casalta H., Frisée V., Moula N., Sartelet A. (2021). Antimicrobial Susceptibility Profile of Several Bacteria Species Identified in the Peritoneal Exudate of Cows Affected by Parietal Fibrinous Peritonitis after Caesarean Section. Vet.-Sci..

[15] Zappa V., Bolaños C.A.D., De Paula C.L., Callefe J.L.R., Alves A.C., De Morais A.B.C., Guerra S.T., Cabrini M.C., Melville P.A., Ribeiro M.G. (2017). Antimicrobial multiple resistance index, minimum inhibitory concentrations, and extended-spectrum beta-lactamase producers of *Proteus mirabilis* and *Proteus vulgaris* strains isolated from domestic animals with various clinical manifestations of infection. Semin. Ciências Agrárias.

[16] Hogan J., Smith K.L. (2003). Coliform mastitis. Vet. Res..

[17] Duse A., Persson-Waller K., Pedersen K. (2021). Microbial Aetiology, Antibiotic Susceptibility and Pathogen-Specific Risk Factors for Udder Pathogens from Clinical Mastitis in Dairy Cows. Animals.

[18] Gupta R., Kumar S., Khurana R. (2020). Essential oils and mastitis in dairy animals: A review. Haryana Vet..

[19] Queiroga M.C., Coelho M.P., Arantes S.M., Potes M.E., Martins M.R. (2018). Antimicrobial Activity of Essential Oils of *Lamiaceae* Aromatic Spices Towards Sheep mastitis-Causing *Staphylococcus aureus* and *Staphylococcus epidermidis*. J. Essent. Oil Bear. Plants.

[20] Szweda P., Zalewska M., Pilch J., Kot B., Milewski S. (2018). Essential oils as potential anti-staphylococcal agents. Acta Vet.-Beogr..

[21] Kovačević Z., Radinović M., Čabarkapa I., Kladar N., Božin B. (2021). Natural Agents against Bovine Mastitis Pathogens. Antibiotics.

[22] Kovačević Z., Kladar N., Čabarkapa I., Radinović M., Maletić M., Erdeljan M., Božin B. (2021). New Perspective of *Origanum vulgare* L. and *Satureja montana* L. Essential Oils as Bovine Mastitis Treatment Alternatives. Antibiotics.

[23] Barreiros Y., de Meneses A.C., Alves J.L.F., Mumbach G.D., Ferreira F.A., Machado R.A.F., Bolzan A., de Araujo P.H.H. (2022). Xanthan gum-based film-forming suspension containing essential oils: Production and in vitro antimicrobial activity evaluation against mastitis-causing microorganisms. LWT.

[24] Tomanić D., Božin B., Čabarkapa I., Kladar N., Radinović M., Maletić M., Kovačević Z. (2022). Chemical Composition, Antioxidant and Antibacterial Activity of Two Different Essential Oils Against Mastitis Associated Pathogens. Acta Vet..

[25] Bassolé I.H.N., Juliani H.R. (2012). Essential Oils in Combination and Their Antimicrobial Properties. Molecules.

[26] Oussalah M., Caillet S., Saucier L., Lacroix M. (2007). Inhibitory effects of selected plant essential oils on the growth of four pathogenic bacteria: *E. coli* O157:H7, *Salmonella* Typhimurium, *Staphylococcus aureus* and *Listeria monocytogenes*. Food Control.

[27] Velebit B., Matekalo-Sverak V., Petrović Z., Lakićević B., Janković V., Lilić S., Vranić D. (2012). Ispitivanje antimikrobne aktivnosti cinamaldehida i karvakrola na mikroorganizme prenosive hranom. Meat Technol..

[28] Bradley A.J., Green M.J. (2001). Adaptation of *Escherichia coli* to the Bovine Mammary Gland. J. Clin. Microbiol..

[29] Bi Y., Wang Y.J., Qin Y., Vallverdú R.G., García J.M., Sun W., Li S., Cao Z. (2016). Prevalence of Bovine Mastitis Pathogens in Bulk Tank Milk in China. PLoS ONE.

[30] Abed A., Menshawy A., Zeinhom M., Hossain D., Khalifa E., Wareth G., Awad M. (2021). Subclinical Mastitis in Selected Bovine Dairy Herds in North Upper Egypt: Assessment of Prevalence, Causative Bacterial Pathogens, Antimicrobial Resistance and Virulence-Associated Genes. Microorganisms.

[31] Di Guardo G., Battisti A., Agrimi U., Forletta R., Reitano M.E., Calderini P. (1997). Pathology of *Serratia marcescens* Mastitis in Cattle. J. Vet.-Med. Ser. B.

[32] Abdullah A.H., Nadhom B.N., Al-Ammiri H.H. (2017). Isolation and Identification of *Serratia marcescens* from Bovine Mastitis infections in Iraq and their Susceptibility to Antibiotics. J. Entomol. Zool. Stud..

[33] Lopes T.S., Fussieger C., Rizzo F.A., Silveira S., Lunge V.R., Streck A.F. (2022). Species identification and antimicrobial susceptibility profile of bacteria associated with cow mastitis in southern Brazil. Pesqui. Vet. Bras..

[34] Ohnishi M., Sawada T., Hirose K., Sato R., Hayashimoto M., Hata E., Yonezawa C., Kato H. (2011). Antimicrobial susceptibilities and bacteriological characteristics of bovine *Pseudomonas aeruginosa* and *Serratia marcescens* isolates from Mastitis. Vet.-Microbiol..

[35] Parmar B., Pal M., Dhami A., Patel J. (2006). Investigation on bovine mastitis caused by *Staphylococcus Aureus*. Int. J. Cow Sci..

[36] Kasa G., Tegegne B., Tadesse B. (2020). Isolation and Identification of Major Pathogenic Bacteria from Clinical Mastitic Cows in Asella Town, Ethiopia. Vet.-Med. Int..

[37] Sumathi B., Veeregowda B., Amitha R.G. (2008). Prevalence and antibiogram profile of bacterial isolates from clinical bovine mastitis. Vet. World.

[38] Verma H., Rawat S., Sharma N., Jaiswal V., Singh R., Harshit V. (2018). Prevalence, bacterial etiology and antibiotic susceptibility pattern of bovine mastitis in Meerut. J. Entomol. Zool. Stud..

[39] Ajose D.J., Oluwarinde B.O., Abolarinwa T.O., Fri J., Montso K.P., Fayemi O.E., Aremu A.O., Ateba C.N. (2022). Combating Bovine Mastitis in the Dairy Sector in an Era of Antimicrobial Resistance: Ethno-veterinary Medicinal Option as a Viable Alternative Approach. Front. Vet.-Sci..

[40] Pascu C., Herman V., Iancu I., Costinar L. (2022). Etiology of Mastitis and Antimicrobial Resistance in Dairy Cattle Farms in the Western Part of Romania. Antibiotics.

[41] Zargoosh Z., Ghavam M., Bacchetta G., Tavili A. (2019). Effects of ecological factors on the antioxidant potential and total phenol content of *Scrophularia striata* Boiss. Sci. Rep..

[42] Türkmen M., Kara M., Maral H., Soylu S. (2021). Determination of chemical component of essential oil of *Origanum dubium* plants grown at different altitudes and antifungal activity against *Sclerotinia sclerotiorum*. J. Food Process. Preserv..

[43] Bozin B., Mimica-Dukic N., Simin N., Anackov G. (2006). Characterization of the Volatile Composition of Essential Oils of Some Lamiaceae Spices and the Antimicrobial and Antioxidant Activities of the Entire Oils. J. Agric. Food Chem..

[44] Kosakowska O., Węglarz Z., Bączek K. (2019). Yield and quality of ‘Greek oregano’ (*Origanum vulgare* L. subsp hirtum) herb from organic production system in temperate climate. Ind. Crop. Prod..

[45] EDQM (2020). European Pharmacopoeia 10.3.

[46] Pyörälä S. (2009). Treatment of mastitis during lactation. Ir. Vet. J..

[47] Vakanjac S., Pavlović V., Magaš V., Pavlović M., Đurić M., Maletić M., Nedić S., Sočo I. (2013). Investigations of efficacy of intramammary applied antimicrobials and glucocorticosteroides in the treatment of subclinical and clinical mastitis in cows. Vet. Glas..

[48] Lavor U.L., Guimarães F.F., Salina A., Mioni M.S., Langoni H. (2019). Bacterial identification, somatic cell count, antimicrobial profile and toxigenic Staphylococcus strains search from mastitic cow milk samples on small farms properties. Pesqui. Veterinária Bras..

[49] Vidović J., Stojanović D., Cagnardi P., Kladar N., Horvat O., Ćirković I., Bijelić K., Stojanac N., Kovačević Z. (2022). Farm Animal Veterinarians’ Knowledge and Attitudes toward Antimicrobial Resistance and Antimicrobial Use in the Republic of Serbia. Antibiotics.

[50] Burović J. (2020). Izolacija bakterijskih patogena kod klinički manifestnih mastitisa mliječnih goveda i njihova antimikrobna osjetljivost u zeničkoj regiji u 2017. godini. Vet. Stanica.

[51] Sun Y., Wen S., Zhao L., Xia Q., Pan Y., Liu H., Wei C., Chen H., Ge J., Wang H. (2020). Association among biofilm formation, virulence gene expression, and antibiotic resistance in Proteus mirabilis isolates from diarrhetic animals in Northeast China. BMC Vet.-Res..

[52] Kayitsinga J., Schewe R., Contreras G., Erskine R. (2017). Antimicrobial treatment of clinical mastitis in the eastern United States: The influence of dairy farmers’ mastitis management and treatment behavior and attitudes. J. Dairy Sci..

[53] Lopes T.S., Fontoura P.S., Oliveira A., Rizzo F.A., Silveira S., Streck A.F. (2020). Use of plant extracts and essential oils in the control of bovine mastitis. Res. Vet.-Sci..

[54] Cerioli M.F., Moliva M.V., Cariddi L.N., Reinoso E.B. (2018). Effect of the Essential Oil of *Minthostachys verticillata* (Griseb.) Epling and Limonene on Biofilm Production in Pathogens Causing Bovine Mastitis. Front. Vet.-Sci..

[55] Tanhaeian A., Sekhavati M.H., Moghaddam M. (2020). Antimicrobial activity of some plant essential oils and an antimicrobial-peptide against some clinically isolated pathogens. Chem. Biol. Technol. Agric..

[56] Fratini F., Casella S., Leonardi M., Pisseri F., Ebani V.V., Pistelli L., Pistelli L. (2014). Antibacterial activity of essential oils, their blends and mixtures of their main constituents against some strains supporting livestock mastitis. Fitoterapia.

[57] Lambert R., Skandamis P., Coote P., Nychas G.-J. (2001). A study of the minimum inhibitory concentration and mode of action of oregano essential oil, thymol and carvacrol. J. Appl. Microbiol..

[58] Adams R.P. (2007). Identification of Essential Oil Components by Gas Chromatography/Mass Spectrometry.

[59] Hudzicki J. (2009). Kirby-Bauer Disk Diffusion Susceptibility Protocol.

[60] CLSI (2008). Performance Standards for Antimicrobial Disk and Dilution Susceptibility Tests for Bacteria Isolated from Animals.

[61] (2012). Performance Standards for Antimicrobial Disk Susceptibility Tests.

[62] CLSI (2018). Methods for Dilution Antimicrobial Susceptibility Tests for Bacteria That Grow Aerobically.

